# An easy intervention to improve short-term adherence to medications in community-dwelling older outpatients. A pilot non-randomised controlled trial

**DOI:** 10.1186/1472-6963-11-158

**Published:** 2011-07-05

**Authors:** Claudio Bilotta, Anna Lucini, Paola Nicolini, Carlo Vergani

**Affiliations:** 1 Department of Internal Medicine, Geriatric Medicine Unit, Fondazione IRCCS Ca' Granda Ospedale Maggiore Policlinico, University of Milan, Milan, Italy; 2 Department of Urban Outpatient Services, Geriatric Medicine Outpatient Service, Istituti Clinici di Perfezionamento Hospital, Milan, Italy; 3 Fondazione Salvatore Maugeri, Istituto Scientifico di Milano, IRCCS, Milan, Italy

## Abstract

**Background:**

Complex interventions to improve compliance to pharmacological treatment in older people have given mixed results and are not easily applicable in clinical practice. The aim of this study was to test the short-term efficacy on self-reported medication adherence of an easy intervention in which the patient or caregiver was asked to transcribe the pharmacological treatment while it was dictated to him/her by the doctor.

**Methods:**

Pilot non-randomised controlled trial involving 108 community-dwelling outpatients aged 65+ (54 in the intervention arm, 54 controls) referred to a geriatric service from May to July 2009 and prescribed by the geriatrician a change in therapy. The intervention was applied at the end of the visit to the person managing the medications, be it the elder or his/her caregiver. Outcome of the study was the occurrence of any adherence error, assessed at a one-month follow-up by means of a semi-structured interview.

**Results:**

The socio-demographic, functional and clinical characteristics of the two compared groups were similar at baseline. At a one-month follow-up 43 subjects (40%) had made at least one adherence error, whether unintentional or intentional. In the intervention group the prevalence of adherence errors was lower than in controls (20% *vs *59%; adjusted odds ratio 0.16, 95% confidence interval 0.07 - 0.39; *p *< 0.001) after adjusting for the person managing the medications, the adherence errors at baseline and for the number of prescribed drugs.

**Conclusions:**

In an older outpatient population the intervention considered was effective in reducing the prevalence of adherence errors in the month following the visit.

**Trial registration:**

Australian and New Zealand Clinical Trials Register (ANZCTR): ACTRN12611000347965

## Background

About 20% of community-dwelling adults aged 65 and older take 10 or more medications daily and polypharmacy in general has been associated with a higher risk of non-adherence to treatment [1,2]. Adherence to treatment can be defined as the extent to which a patient's actual drug regimen - in terms of dosage, time and mode of administration of the drugs - corresponds to the prescriptions made by the doctor [3]. In older people a poor adherence to pharmacological treatment has been reported in 26 - 59% of cases, depending on the population considered and on the operational definition of adherence adopted in different clinical trials [4-9], and is associated with a decline in clinical status, a greater risk of falls, hospitalisation and death as well as an increase in health expenditures [4,10].

The main barriers to adherence in elders are forgetting to take the drug, limited organisational skills, belief that the drug is ineffective or unnecessary, and costs [1]. With reference to organisational skills, a study involving an elderly population has highlighted that about 22% of those who had physical disabilities and about one third of those affected by cognitive impairment actually managed their own medications [11]. Another study has found that about half of the elders had difficulties in understanding the instructions concerning drug therapy [12].

Adherence can be improved by providing caregivers to elders who are unable to take their medications properly and by encouraging doctors to routinely review their patients' pharmacological treatment in order to ensure that drug regimens are adequate and as simple as possible [1,2,13]. Moreover, there is evidence that a better adherence can be obtained if patients (and caregivers) are engaged in decisions about starting or keeping or changing a chronic therapy [9]; in the specific case of frail older patients it has been shown that their desire for involvement is essentially fulfilled by good communication and information and does not extend to the decision-making process [14].

Nevertheless, according to recent reviews, clinical trials to determine the efficacy of a range of different interventions on the adherence to treatment of older subjects seem to offer a rather bleak outlook: results are mixed and the average increase in adherence in the intervention group compared to the control group is low (11%) [13,15-18]. It must however be emphasised that these clinical trials have a number of limitations: the interventions tested are complex and expensive and therefore difficult to apply to everyday clinical practice; almost all interventions combine more than one strategy and it is not possible to evaluate the effects on adherence of their individual components; there is often little involvement of the patient who is thus confined to a mainly passive role; adherence to treatment is high even in the control groups, probably because patients derive motivation from the mere awareness they are participating in a research study on adherence; baseline adherence is already high because patients affected by cognitive impairment, at risk of non-adherence, are often excluded [15-18].

We hypothesize that a simple intervention at the end of the visit - i.e. the transcription by the patient or caregiver of the pharmacological treatment dictated to him/her by the doctor - can be valuable in improving patients' adherence to therapy, at least in the short term. Such intervention has the potential to be effective both psychologically and organisationally. Psychologically because it can enhance the sense of empowerment of patients/caregivers by allowing them to play a more active role in the health-care process. Organisationally because it results in a medication schedule in which the dosages and the times of administration of the prescribed drugs have been clearly noted under the doctor's supervision: this can avoid the mistakes that occur when, on their own, the patients or caregivers copy the pharmacological treatment from the medical report into an informal scheme for personal use and can also offer the doctor a further opportunity to dissipate misunderstandings and eventually tailor the treatment to the patients' daily routine.

Aim of the study was therefore to assess the short-term (i.e. one month) efficacy of an intervention of this kind on the self-reported adherence to pharmacological treatment in a sample of older community-dwelling subjects referred to an outpatient geriatric service in Milan, Italy.

## Methods

### Design, setting and participants

**Figure 1:**
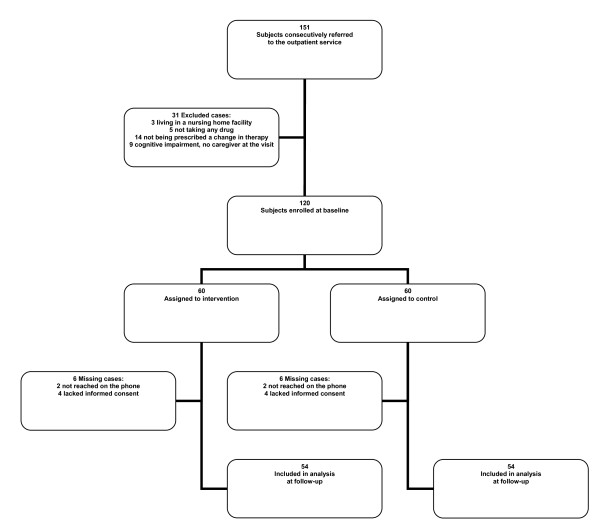
**Study participants, their allocation and follow-up**.

This pilot non-randomised controlled, parallel trial considered for inclusion a sample of 151 older people (i.e. aged 65+) consecutively referred to the outpatient geriatric service of the Fondazione IRCCS Ca' Granda Ospedale Maggiore Policlinico in Milan, Italy, from May 2^nd ^to July 31^st ^2009. Patients were referred by their general practitioners (GPs) in order to receive a comprehensive geriatric assessment and medical advice for several reasons including functional decline, falls, weight loss, cognitive decline, depression, and management of multi-drug therapy. Patients were selected at the end of their visits according to the following inclusion criteria: living in the community, taking at least one drug a day, having been prescribed a change in pharmacological treatment during the visit (i.e. at least one drug was added or suspended by the doctor) and - in the case of patients dependent in the management of medications - having with them at the visit the caregiver who was in charge of the administration of drugs. According to these inclusion criteria 120 subjects were enrolled in the study (Figure 1). The study protocol was designed and performed according to the code of ethics of the Declaration of Helsinki. All patients and caregivers (if included, see next paragraph) gave written informed consent to participation in the study.

### Baseline comprehensive geriatric assessment

All patients during their visits underwent a comprehensive geriatric evaluation, performed by a multi-professional team including two geriatricians, from which baseline measures were retrieved. The main socio-demographic characteristics of the older participants (age, gender, years of schooling and living arrangements) were considered and detailed information on medical history was also collected. Functional status was assessed by means of the Basic Activities of Daily Living (BADL) [19] and Instrumental Activities of Daily Living (IADL) [20] scales, cognitive status by means of the Mini-Mental State Examination (MMSE) scale with score correction for age and education [21], emotional status by means of the 5-item Geriatric Depression Scale (GDS) [22] in subjects without dementia and the Cornell scale for depression in dementia in the remaining subjects [23], comorbidity by means of the Cumulative Illness Rating Scale morbidity (CIRS-m) and severity (CIRS-s) scales [24], nutritional status by means of the Mini Nutritional Assessment Short Form (MNA-SF) [25].

The caregiver, either informal or formal, was also recruited if he/she was responsible for the provision of drug therapy. We considered the following characteristics of caregivers: age, years of schooling and levels of distress quantified by means of the Caregiver Burden Inventory scale (CBI) [26]. With regard to the pharmacological treatment prescribed during the visit we considered different variables: the number of specific drugs to be taken daily, the number of overall drug administrations per day and the classes of drugs involved.

At the baseline visit the two geriatricians involved in the CGA asked the patients and/or their caregivers if they were willing to give their consent to answering a brief semi-structured interview on the adherence to pharmacological treatment in the preceding month. The semi-structured interview was administered to the person who managed the medications and inquired about possible adherence errors, classified as 'unintentional' or 'intentional'. The former are not deliberate, i.e. they are made by subjects who wish to follow the doctor's prescriptions but are for some reason unable to do so properly; they can, for instance, be ascribed to forgetfulness, misunderstanding or temporary lack of access to the drug (running out of medication supplies or not being at home at the time the drug should be taken). The latter are deliberate, i.e. they are made by subjects who intentionally decide not to follow the prescribed regimen due to misconceptions about illness and medication; they derive from personal beliefs regarding the need, effectiveness and safety of drugs and include not taking a prescribed drug (never starting the treatment or stopping it) or, conversely, not suspending a drug deemed inappropriate or potentially harmful by the doctor. During the interview participants (patients or caregivers) were asked to focus on the month prior to the visit and indicate for each of the drugs listed in the referral letter from their GP if i) it had been taken/given correctly in terms of dosages and times of administration, ii) it had not been taken/given correctly because of forgetfulness, misunderstanding or temporary unavailability, iii) it had not been taken/given correctly because of a deliberate choice to never take it/give it or to suspend it. Moreover, patients/caregivers were asked if they were taking/giving drugs not prescribed by the doctor or that the doctor had decided to suspend. Since subjects are often reluctant to admit to non-adherence [9], possible errors were investigated in a tactful and neutral manner so that patients/caregivers would not feel their conduct was being judged (e.g. '*We know it is hard to take all these medicines regularly, if you look back at the last month has it ever happened that you missed a dose of a drug ?'; 'In the last month did you make any adjustment to the drug regimen prescribed by the doctor because you felt uncomfortable with it?')*.

### Intervention

The two geriatricians involved in the baseline visit were blinded to the allocation of participants. At the end of the visit, a third geriatrician - who had ready access to the information noted by his colleagues in the medical record - selected participants according to the inclusion criteria, alternately allocated them to the intervention or control groups (allocation ratio 1:1) and administered the intervention after having described it to the participants and having obtained a written consent to it. Thus at the end of the visit half of the subjects (*n *= 60) fulfilling inclusion criteria underwent a quick and easy intervention with the objective to improve their adherence to pharmacological treatment. It was applied to the person managing the medications (patient or caregiver) who was asked to write down on a sheet of paper the drug regimen that had been prescribed (i.e. all prescribed medications and not only new/changed medications). This was dictated to him/her by the doctor to obtain a schedule of pharmacological treatment in which the times of administration of the drugs were noted along with their dosages. The doctor supervised the operation, making sure that the transcription was correct and clear, and the patient or caregiver was then given the schedule for use at home. The other half of the study population received no such intervention but only the standard procedure available at our outpatient geriatric service: the prescribed pharmacological treatment was stated at the end of the medical report and reviewed orally by the geriatrician performing the visit with the person in charge of medications. The patient or caregiver was then given a copy of the medical report containing the medication instructions. All the doctors taking part in the study were adequately trained so as to ensure they offered patients an approach that would be as much as possible uniform. In particular they were instructed on how to carefully collect information on pharmacological treatment at baseline, how to tactfully inquire about possible adherence errors in the month prior to the visit, how to orally review the prescribed therapy in an accurate manner and - for the intervention group - how to involve patients/caregivers in the dictation procedure. In order to verify the consistency of the doctors' behaviour and to estimate the duration of the intervention, for the first 40 cases recruited (20 in the intervention arm, 20 controls) an independent medical investigator was also present at the visit and during the intervention - when this was performed - to function as a supervisor.

### One-month follow-up

One month after the visit the person managing the medications (patient or caregiver) was called on the phone by another investigator, who had not been involved in the preceding steps and was therefore blinded to baseline adherence and group assignment, in order to assess the adherence to the pharmacological treatment prescribed at the visit (i.e. written by the doctor in the medical report of the visit). Outcome of the study was taken to be the presence of at least one self-reported adherence error of any kind (i.e. unintentional or intentional) during this period. Errors were evaluated by means of the same semi-structured interview used at baseline (see Baseline comprehensive geriatric assessment). One month was considered an appropriate time interval for a short-term follow-up, in accordance with the inclusion criteria of a recent review of randomised clinical trials aimed at improving medication adherence in community-dwelling elders [15]. In order to avoid a potential bias (i.e. an artificially low prevalence of adherence errors in both groups) participants were not told in advance they would be given a subsequent telephone interview to evaluate their adherence to treatment; it is a standard procedure of the visits at our outpatient geriatric service to ask patients and/or caregivers if they consent to leaving telephone numbers at which they can be reached if necessary. All the participants who were contacted on the phone after one month were mailed an informed consent form for the treatment of the personal data obtained at follow-up and were invited to fill it in and send it back to us. Unfortunately, 12 patients were lost to follow-up: four (two in each group) could not be contacted on the phone and eight (four in each group) of those who were contacted lacked informed consent. The study sample was therefore composed of 108 patients (Figure 1). The demographic characteristics of the 12 patients lost to follow-up were consistent with those of the recruited sample.

### Statistical analyses and sample size calculations

The main baseline characteristics of the sample were analysed by calculating means and standard deviations for metric variables and percentages for nominal variables. The comparison between the two groups - intervention group and control group - was carried out using Student's t-test for metric variables with a normal distribution and the chi-squared test or Fisher's exact test for nominal variables. In order to assess the efficacy of the intervention after one month, a multivariate logistic regression analysis was conducted with the study outcome (see previous section) as the dependent variable and having received the intervention as the covariate; corrections were made for adherence errors at the baseline evaluation, number of prescribed drugs (i.e. highest tertile *vs *rest) and for the person who managed the medications (i.e. patient or caregiver). A p value < 0,05 was assumed to indicate statistical significance. Statistical analyses were performed by means of the statistical package SPSS 14.0 for Windows.

As far as the sample size was concerned, since ours was meant to be a pilot study and loss to follow-up was assumed to be negligible because of the short duration of the follow-up (one month), we recruited 120 subjects at the baseline evaluation in order to have 60 subjects in each group. Even though a few participants were actually lost at follow-up, it was calculated that a sample of 54 cases per group had a statistical power of 80% at a 5% alpha level to detect an absolute decrease of 23% of non-adherence to medication in the intervention group, assuming nearly 50% of non-adherence in the control group [8].

## Results

### Baseline evaluation: characteristics of the sample

The sample was composed of 108 subjects with a mean age of 80 (range 65-95), 66% (*n *= 71) of whom were women. Thirty-seven percent (*n *= 40) of patients were affected by cognitive impairment and 67% (*n *= 72) suffered from symptoms of depression. Forty-nine participants (45%) had more than five chronic diseases, i.e. a comorbidity index greater than 5 at the CIRS. With regard to the pharmacological treatment prescribed at the visit, patients had to take an average of 5.5 (SD 2.6) drugs per day and the mean number of daily drug administrations was 6.5 (SD 3.3). The most commonly prescribed drugs were: antiaggregants/anticoagulants (69%), antihypertensives (69%), gastroprotectives (44%) and antidepressants (42%). In 40% of cases (*n *= 43) it was the caregiver who managed the medications.

In 69% (*n *= 74) of the sample we observed at least one adherence error of any kind in the month prior to the visit: 28% (*n *= 30) of the participants made only unintentional adherence errors, 24% (*n *= 26) made only intentional adherence errors, while 17% (*n *= 18) made both types of errors.

The baseline profile of the 108 patients belonging to the intervention and control groups is summarised in Table 1: the two groups were similar in terms of socio-demographic data, functional status, comorbidity, polipharmacy and prevalence of non-adherence errors in the past month as well as characteristics of the caregivers (when present). The median duration of the intervention, assessed for the first 20 cases assigned to the intervention group, was 3 minutes (range 1 to 5 minutes).

**Table 1:** Baseline characteristics of the 108 older participants and their 43 caregivers.

Variables	Intervention (*n *= 54)		Control (*n *= 54)		*P *value
	Mean (SD)	Perc. (*n*)	Mean (SD)	Perc. (*n*)	
Older participants					
Age, years	79.8 (6.4)		80.6 (7.1)		0.543
Sex: female		63 (34)		69 (37)	0.543
Living alone		50 (27)		44 (24)	0.780
Education, years	8.8 (5)		8.5 (4.4)		0.698
BADL score^a^	4.6 (1.6)		4.4 (1.5)		0.623
IADL score^b^	4.6 (2.9)		4.3 (2.7)		0.495
Dependence in taking medications		35 (19)		44 (24)	0.326
CIRS m score^c^	4.2 (1.8)		4.1 (1.6)		0.610
CIRS s score^d^	1.9 (0.5)		1.9 (0.3)		0.543
MMSE score^e^	24.1 (6.3)		23.7 (6.3)		0.726
5-item GDS score^f^	2.4 (1.3)		2.0 (1.3)		0.162
Cornell scale score^g^	13.4 (5.2)		15.0 (5.1)		0.344
MNA SF score^h^	10.4 (2.1)		9.7 (2.4)		0.132
Drugs daily, *n*	5.5 (2.6)		5.4 (2.6)		0.854
Drug administrations daily, *n*	6.7 (3.6)		6.4 (3.1)		0.668
Any adherence error^i^		70 (38)		67 (36)	0.679
Any intentional adherence error		41 (22)		41 (22)	1.000
Therapy suspended		33 (18)		26 (14)	0.399
Therapy never taken		11 (6)		19 (10)	0.417^†^
Therapy never stopped		2 (1)		13 (7)	0.060^†^
Any unintentional adherence error		52 (28)		37 (20)	0.121
Caregivers	(*n *= 19)		(*n *= 24)		
Age, years	62.8 (16)		58.1 (18.9)		0.380
Education, years	7.5 (3.3)		7.6 (3.8)		0.955
CBI score^l^	35.2 (22.1)		37.1 (20.9)		0.766

^† ^Statistical analysis was performed by using Fisher's exact test.

^a ^BADL = Basic Activities of Daily Living; normal score 6/6. Lower scores indicate worse functional status.

^b ^IADL = Instrumental Acitivities of Daily Living; normal score 8/8. Lower scores indicate worse functional status.

^c ^CIRS m = Cumulative Illness Rating Scale morbidity. Score range 0-13; lower scores indicate lower morbidity.

^d ^CIRS s = Cumulative Illness Rating Scale severity. Score range 1-5; lower scores indicate less severe multiple pathology.

^e ^MMSE = Mini Mental State Examination. Score range 0-30. Scores are corrected for age and education. Lower scores indicate worse cognitive function.

^f ^GDS = Geriatric Depression Scale. Score range 0-5. Higher scores indicate worse emotional status. This variable was analysed only in the 68 participants (34 per group) without cognitive impairment.

^g ^Score range 0-38, higher scores indicate worse emotional status. This variable was analysed only in the 40 participants (20 per group) suffering from cognitive impairment (i.e. MMSE score < 24 out of 30 after correction for age and education).

^h ^MNA SF = Mini Nutritional Assessment Short Form. Score range 0-14. Lower scores indicate higher risk of malnutrition.

^i ^The sum of any intentional and any unintentional errors is over 100% because we found both kinds of errors in 18 cases: 12 cases belonging to the intervention group and 6 cases belonging to the control group.

^l ^CBI = Caregiver Burden Inventory. Score range 0-96, higher scores indicate greater distress of the caregiver.

### One-month follow-up

At the follow-up, carried out one month after the visit, we found that 43 subjects (40%) had made at least one adherence error, whether unintentional or intentional, in this interval of time. The prevalence of adherence errors was 20% (*n *= 11) in the intervention group and 59% (*n *= 32) in the control group (*p *< 0.001) (Table 2). At multivariate logistic regression analysis the intervention tested was associated with a lower risk of making errors of any kind in the adherence to pharmacological treatment when compared to the control group (odds ratio 0.16, 95% confidence interval 0.06 - 0.39; *p *< 0.001), after adjusting for previous adherence errors (i.e. prevalence of adherence errors at the baseline evaluation) and for the person who was in charge of the medications (i.e. patient or caregiver) (Table 3 model a). The results of the multivariate analysis were unchanged even after adjusting for a large number of drugs taken (Table 3 model b).

**Table 2:** Non-adherence to medications at a one-month follow-up.

Outcomes	Intervention (*n *= 54)	Control (*n *= 54)	*P *value
	Perc. (*n*)	Perc. (*n*)	
Any adherence error	20 (11)	59 (32)	< 0.001
Therapy suspended	13 (7)	33 (18)	0.021^†^
Therapy never taken	7 (4)	28 (15)	0.010^†^
Therapy never stopped	0 (0)	19 (10)	0.001^†^
Unintentional adherence error	15 (8)	32 (17)	0.067^†^

^† ^Statistical analysis was performed by using Fisher's exact test.

**Table 3:** Logistic regression analysis of non-adherence to medications at follow-up (*n *= 108).

Variables	Adjusted OR	95% CI	*P *value
Model a)			
Educational intervention	0.16	0.06 - 0.39	< 0.001
Baseline characteristics			
Any intentional adherence error	1.62	0.68 - 3.86	0.276
Any unintentional adherence error	1.29	0.53 - 3.15	0.572
Therapy managed by the caregiver	0.71	0.29 - 1.74	0.452
			
Model b)^†^			
Educational intervention	0.16	(0.07 - 0.39)	< 0.001

† Model adjusted even for a large number of prescribed drugs (highest tertile *vs *rest, i.e. 7+ drugs *vs *rest) besides the baseline covariates of Model a.

## Discussion

The intervention assessed in our study - i.e. the transcription by the patient/caregiver of the pharmacological treatment dictated to him/her by the doctor at the end of the visit - was found to be effective since there was a significantly lower prevalence of adherence errors in the intervention group (20%) than in the control group (59%) in the month following the visit. The improvement in adherence was confirmed after adjusting for the adherence at baseline, the number of prescribed drugs and the person to whom the intervention was directed (patient or caregiver, according to who managed the medications). The relevance of these findings is enhanced by the fact that the intervention was demonstrated to be effective in a population at high risk of non-adherence - older subjects who had been prescribed a change in their usual pharmacological treatment and who had to take an average of five drugs per day - and that it was tested against a control group who underwent a standard procedure which is itself very much focused on improving adherence. In fact it is part of the geriatrician's routine practice to simplify and personalise the drug regimens of the older patients and to make sure that all instructions are clear and understood. Indeed even in the control group the prevalence of adherence errors was slightly reduced - from 67% to 59% - from baseline to follow-up.

Our study shows a greater increase in adherence from the intervention to the control group than most studies on adherence carried out in older populations (please see Background). This could be because in the intervention we tested, unlike in others, the patient/caregiver who manages the medications is not just a passive recipient of information but is made to play a more active role in the partnership with the physician, thus enhancing his/her motivation. It is acknowledged that adherence to therapy is also a matter of personal choice [16]. Moreover, the transcription of therapy engages two sensory channels (i.e. listening and writing) while the standard procedure only one (i.e. listening) and this could favour the learning process. In addition, a medication schedule in which the dosages and the times of administration of the drugs are written under the doctor's supervision can hold several advantages: prevention of transcription errors and the opportunity to clear misunderstandings and tailor the treatment to the patient's daily routine.

Another point deserving further discussion is that the prevalence of adherence errors observed in the control group after the visit is relatively higher than is reported in literature [4-9], notwithstanding the short duration of our follow-up (one month). A possible explanation lies in the selection of a sample at high risk of non-adherence (the patients considered were those who had been prescribed a change in the pharmacological treatment during the visit), in having considered both intentional and unintentional medication errors, and in the avoidance of a potential bias due to the awareness that adherence would be subsequently re-evaluated. In fact patients and caregivers were not told in advance they would be given a phone interview after one month and this was a methodological choice which distinguishes our study from most other studies and could constitute one of its strengths.

There are several other strengths to our study: we analysed adherence to the complex multi-drug regimens that are the rule in older subjects, while many studies have considered younger subjects and/or adherence to a specific class of medications [15,16,27-29]; we tested a single intervention easy to apply to everyday clinical practice whereas many studies have tested complex and combined strategies, often administered by appropriately trained health-care professionals like nurses and pharmacists, whose routine application would be more cumbersome [16,30-35]; we assessed the efficacy of our intervention both on patients and on caregivers (when they were in charge of the medications).

The study also has some limitations. Having been conceived as a pilot study, the main ones are the fact that the geriatrician who administered the intervention was not blinded to group assignment, the relatively small sample size and the short duration of the follow-up period. The limited sample size did not allow us to separately evaluate the effect of the intervention on the two types of adherence error (i.e. unintentional or intentional). The short duration of the follow-up enabled us to assess the efficacy of the intervention only in the short-term. Furthermore, this study was not a randomised one according to CONSORT guidelines, since participants were alternately allocated to the intervention or control groups. However we have to underline that the socio-demographic, functional and clinical characteristics, including the number of prescribed drugs, of the two compared groups were similar at baseline. Further randomised controlled studies will therefore be necessary to confirm the efficacy of the intervention on a larger sample of community-dwelling older people and to extend the investigation to longer follow-up periods (mid-term and long-term).

Some mention must also be made of a key methodological issue: the outcome of the study (i.e. the occurrence of any adherence error during a one-month period) was evaluated by means of a semi-structured phone interview. Although this approach suffers from the limitations imposed by self-reporting, such as the recall and social desirability biases (i.e. not remembering or not admitting errors) we still believe it is a reasonable choice when compared to other options like structured interviews (e.g. the Morisky scale) and Medication Event Monitoring Systems (MEMSs). The Morisky scale not only exhibits the same limitations due to self-reporting but also does not consider, even in its most recent expanded version [36], some of the intentional errors investigated by our semi-structured interview, like never starting the treatment or not suspending a drug deemed inappropriate or potentially harmful by the doctor. MEMSs certainly provide an objective and reliable measure of adherence but, since patients are necessarily aware they are being monitored, these devices are known to improve adherence in the short-term [37,38]; the use of MEMSs would have thus introduced an important bias in a study focusing on adherence in the first month after an intervention.

We included in the study subjects who had been prescribed a change in the pharmacological treatment during the visit (i.e. those at higher risk of non-adherence) and excluded subjects whose drug regimens were unchanged (i.e. those at lower risk of non-adherence). This choice was made in order to ensure that the sample considered would be as homogeneous as possible in terms of the risk of non-adherence and to test the efficacy of the intervention in the most challenging adherence scenario.

In our study patients unable to take their medications properly were included only if they had a caregiver who was responsible for the provision of therapy. A recent study carried out in Germany has shown that among older outpatients suffering from Alzheimer's disease compliance to antidementia drugs, such as donepezil and memantine, was relatively high, but drug therapy was supervised or completely managed by the carers in 94% of participants [39]. Therefore a suggestion for further research can be addressing the issue of adherence in dependent subjects lacking assistance.

## Conclusions

In an older outpatient population referred to our geriatric service the transcription by the patient/caregiver of the pharmacological treatment dictated to him/her by the doctor at the end of the visit was effective in significantly reducing the prevalence of adherence errors in the month following the visit. The intervention tested in this study, simple and easy to apply, could be routinely used in everyday clinical practice to improve medication adherence, at least in the short-term, among community-dwelling elders and their caregivers.

## Competing interests

The authors declare that they have no competing interests.

## Authors' contributions

CB was responsible for the data, contributed to the literature review and study design, was involved in data collection, performed statistical analyses and drafted the manuscript; AL was responsible for the data, contributed to the literature review and study design, was involved in data collection and statistical analyses; PN was involved in data collection and revised the manuscript; CV was responsible for the data, contributed to the literature review and revised the manuscript. All authors read and approved the final manuscript.

## Pre-publication history

The pre-publication history for this paper can be accessed here:

http://www.biomedcentral.com/1472-6963/11/158/prepub

## References

[B1] Steinman MA Hanlon JT Managing medications in clinically complex elders. "There's got to be a happy medium" JAMA 2010 304 1592 1601 10.1001/jama.2010.1482 20940385 PMC2981606

[B2] Vik SA Maxwell CJ Hogan DB Measurement, correlates, and health outcomes of medication adherence among seniors Ann Pharmacother 2004 38 303 12 14742770 10.1345/aph.1D252

[B3] Vermeire E Hearnshaw H Van Royen P Denekens J Patient adherence to treatment: three decades of research: a comprehensive review J Clin Pharm Ther 2001 26 331 42 10.1046/j.1365-2710.2001.00363.x 11679023

[B4] Berry SD Quach L Procter-Gray E Kiel DP Li W Samelson EJ Lipsitz LA Kelsey JL Poor adherence to medications may be associated with falls J Gerontol Med Sci 2010 65 553 8 10.1093/gerona/glq027 PMC2854886 20231214

[B5] Borah B Sacco P Zarotsky V Predictors of adherence among Alzheimer's disease patients receiving oral therapy Curr Med Res Opin 2010 26 1957 65 10.1185/03007995.2010.493788 20569067

[B6] Cardenas-Valladolid J Martin-Madrazo C Salinero-Fort MA de-Santa Pau EC Abanades-Herranz JC de Burgos-Lunar C Prevalence of adherence to treatment in homebound elderly people in primary health care: a descriptive, cross-sectional, multicentre study Drugs Aging 2010 27 641 51 10.2165/11537320-000000000-00000 20658792

[B7] Setoguchi S Choudhry NK Levin R Shrank WH Winkelmayer WC Temporal trends in adherence to cardiovascular medications in elderly patients after hospitalization for heart failure Clin Pharmacol Ther 2010 88 548 54 10.1038/clpt.2010.139 20827266

[B8] Van Eijken M Tsang S Wensing M de Smet PA Grol RP Interventions to improve medication compliance in older patients living in the community: a systematic review of the literature Drugs Aging 2003 20 229 40 10.2165/00002512-200320030-00006 12578402

[B9] Osterberg L Blaschke T Adherence to medication N Engl J Med 2005 353 487 97 10.1056/NEJMra050100 16079372

[B10] Vik SA Hogan DB Patten SB Johnson JA Romonko-Slack L Maxwell CJ Medication nonadherence and subsequent risk of hospitalisation and mortality among older adults Drugs Aging 2006 23 345 56 10.2165/00002512-200623040-00007 16732693

[B11] Tafreshi MJ Melby MJ Kaback KR Nord TC Medication-related visits to the emergency department: a prospective study Annals Pharmacother 1999 33 1252 7 10.1345/aph.19062 10630823

[B12] Beijer HJM de Blaey CJ Hospitalisations caused by adverse drug reactions (ADR): a meta-analysis of observational studies Pharm World Sci 2002 24 46 54 10.1023/A:1015570104121 12061133

[B13] van Dulmen S Sluijs E van Dijk L de Ridder D Heerdink R Bensing J Patient adherence to medical treatment: a review of reviews BMC Health Serv Res 2007 7 55 10.1186/1472-6963-7-55 17439645 PMC1955829

[B14] Ekdahl AW Andersson L Friedrichsen M "They do what they think is the best for me." Frail elderly patients' preferences for participation in their care during hospitalization Patient Educ Couns 2010 80 233 40 10.1016/j.pec.2009.10.026 19945814

[B15] George J Elliott RA Stewart DC A systematic review of interventions to improve medication taking in elderly patients prescribed multiple medications Drugs Aging 2008 25 307 24 10.2165/00002512-200825040-00004 18361541

[B16] Higgins N Regan C A systematic review of the effectiveness of interventions to help older people adhere to medication regimes Age Ageing 2004 33 224 9 10.1093/ageing/afh072 15082425

[B17] Conn VS Hafdahl AR Cooper PS Ruppar TM Mehr DR Russel CL Interventions to improve medication adherence among older adults Gerontologist 2009 49 447 62 10.1093/geront/gnp037 19460887

[B18] Kripalani S Yao X Haynes RB Interventions to enhance medication adherence in chronic medical conditions Arch Intern Med 2007 167 540 50 10.1001/archinte.167.6.540 17389285

[B19] Katz S Downs TD Cash HR Grotz RC Progress in development of the index of ADL Gerontologist 1970 1 20 3 10.1093/geront/10.1_part_1.20 5420677

[B20] Lawton MP Brody EM Assessment of older people: self-maintaining and instrumental activities of daily living Gerontologist 1969 9 179 86 10.1093/geront/9.3_Part_1.179 5349366

[B21] Folstein MF Folstein SE McHugh PR "Mini-mental state": a practical method for grading the cognitive state of patients for the clinician J Psychiatr Res 1975 12 189 98 10.1016/0022-3956(75)90026-6 1202204

[B22] Hoyl MT Alessi CA Harker JO Josephson KR Pietruszka FM Koelfgen M Mervis JR Fitten LJ Rubenstein LZ Development and testing of a five-item version of the Geriatric Depression Scale J Am Geriatr Soc 1999 47 873 8 10404935 10.1111/j.1532-5415.1999.tb03848.x

[B23] Alexopoulos GS Abrams RC Young RC Shamoian CA Cornell scale for depression in dementia Biol Psychiatry 1988 23 271 84 10.1016/0006-3223(88)90038-8 3337862

[B24] Parmelee PA Thuras PD Katz IR Lawton MP Validation of the Cumulative Illness Rating Scale in a geriatric residential population J Am Geriatr Soc 1995 43 130 7 7836636 10.1111/j.1532-5415.1995.tb06377.x

[B25] Rubenstein LZ Harker JO Salvà A Guigoz Y Vellas B Screening for undernutrition in geriatric practice: developing the short-form mini-nutritional assessment (MNA-SF) J Gerontol Med Sci 2001 56 M366 M372 10.1093/gerona/56.6.M366 11382797

[B26] Novak M Guest C Application of a multidimensional Caregiver Burden Inventory Gerontologist 1989 29 798 800 10.1093/geront/29.6.798 2516000

[B27] Vervloet M van Dijk L Santen-Reestman J van Vlijmen B Bouvy ML de Bakker DH Improving medication adherence in diabetes type 2 patients through Real Time Medication Monitoring: a randomised controlled trial to evaluate the effect of monitoring patients' medication use combined with short message service (SMS) reminders BMC Health Serv Res 2011 11 5 10.1186/1472-6963-11-5 21219596 PMC3024217

[B28] Bosworth HB Olsen MK Neary A Orr M Grubber J Svetkey L Adams M Oddone EZ Take control of your blood pressure (TCYB) study: a multifactorial tailored behavioural and educational intervention for achieving blood pressure control Patient Educ Couns 2008 70 338 47 10.1016/j.pec.2007.11.014 18164894 PMC2276731

[B29] Lau R Stewart K McNamara KP Jackson SL Hughes JD Peterson GM Bortoletto DA McDowell J Bailey MJ Hsueh A George J Evaluation of a community pharmacy-based intervention for improving patient adherence to antihypertensives: a randomised controlled trial BMC Health Serv Res 2010 10 34 10.1186/1472-6963-10-34 20137091 PMC2829019

[B30] Esposito L The effects of medication education on adherence to medication regimens in an elderly population J Adv Nursing 1995 21 935 43 10.1046/j.1365-2648.1995.21050935.x 7602002

[B31] Lipton HL Bird JA The impact of clinical pharmacists' consultations on geriatric patients' compliance and medical care use: a randomised controlled trial Gerontologist 1994 34 307 15 10.1093/geront/34.3.307 8076871

[B32] Lowe CJ Raynor DK Purvis J Farrin A Hudson J Effects of a medicine review and education programme for older people in general practice Br J Clin Pharmacol 2000 50 172 5 10.1046/j.1365-2125.2000.00247.x 10930970 PMC2014400

[B33] Ware GJ Holford NH Davison JG Harris RG Unit dose calendar packaging and elderly patient compliance N Z Med J 1991 104 495 7 1745461

[B34] Nazareth I Burton A Shulman S Smith P Haines A Timberall H A pharmacy discharge plan for hospitalised elderly patients - a randomised controlled trial Age Ageing 2001 30 33 40 10.1093/ageing/30.1.33 11322670

[B35] Wolfe SC Schirm V Medication counseling for the elderly: effects on knowledge and compliance after hospital discharge Geriatr Nursing 1992 13 134 8 10.1016/S0197-4572(07)81022-6 1319935

[B36] Morisky DE Ang A Krousel-Wood MA Ward KJ Predictive validity of a medication adherence measure in an outpatient setting J Clin Hypertens 2008 10 348 54 10.1111/j.1751-7176.2008.07572.x PMC2562622 18453793

[B37] Rosen MI Rigsby MO Salahi JT Ryan CE Cramer JA Electronic monitoring and counselling to improve medication adherence Behav Res Ther 2004 42 409 22 10.1016/S0005-7967(03)00149-9 14998735

[B38] Wetzels GE Nelemans PJ Schouten JS Dirksen CD van der WT Stoffers HE Janknegt R de Leeuw PW Prins MH Electronic monitoring of adherence as a tool to improve blood pressure control. A randomised controlled trial Am J Hypertens 2007 20 119 25 10.1016/j.amjhyper.2006.07.018 17261454

[B39] Schwalbe O Scheerans C Freiberg I Schmidt-Pokrzywniak A Stang A Kloft C Compliance assessment of ambulatory Alzheimer patients to aid therapeutic decisions by healthcare professionals BMC Health Serv Res 2010 10 232 10.1186/1472-6963-10-232 20696034 PMC2928215

